# Mothers/caregivers healthcare seeking behavior towards childhood illness in selected health centers in Addis Ababa, Ethiopia: a facility-based cross-sectional study

**DOI:** 10.1186/s12887-019-1588-2

**Published:** 2019-07-03

**Authors:** Nebiat Teshager Abegaz, Hailemariam Berhe, Gebremedhin Beedemariam Gebretekle

**Affiliations:** 1 Ethiopian Public Health Association, Addis Ababa, Ethiopia; 2 Department of Nursing, Mekelle University, Mekelle, Ethiopia; 3 Department of Pharmaceutics and Social Pharmacy, School of Pharmacy, College of Health Sciences, Addis Ababa University, Addis Ababa, Ethiopia

**Keywords:** Healthcare seeking behavior, Childhood illness, Mothers/caregivers, Under-five children, Addis Ababa, Ethiopia

## Abstract

**Background:**

Seeking healthcare in children is unique since parents decide upon the type and frequency of healthcare services accessed. Mothers/caregivers lower healthcare seeking behavior is one of the major reason for increased morbidity and mortality from childhood illness in developing countries. Hence, this study aimed to assess healthcare seeking behavior of mothers/caregivers towards childhood illnesses in selected health centers of Addis Ababa, Ethiopia.

**Methods:**

A facility-based cross-sectional survey was conducted on 422 sampled mothers/caregivers of children age 0–59 months, from April 18 to May 11, 2016. Ten health centers were selected using simple random sampling technique and proportionate number of participants were included from each health centers. A pre-tested, semi-structured questionnaire was used to collect data. Data were analyzed using SPSS version 20.0. Descriptive statistics was used to summarize socio-demographic characteristics and multivariable logistic regression was employed to identify factors associated with of healthcare seeking behavior.

**Result:**

In case of illnesses, 26.5% of mothers/caregivers sought healthcare for their children. Among the common childhood illnesses, acute respiratory tract infection and diarrhea accounted for 47.6 and 31%, respectively. Mothers/caregivers healthcare seeking behavior towards common childhood illnesses were influenced by child’s age (AOR = 1.78, 95% CI:1.02, 3.13), education of mothers/caregivers (AOR = 4.24, 95% CI:1.32, 13.63), family size (AOR = 3.83, 95% CI:1.06, 13.78), perception of severity of illnesses (AOR = 2.00, 95% CI:1.05, 3.84), previous experience of similar illnesses (AOR = 3.67, 95% CI:1.36, 9.86) and previous history of under-five child death (AOR = 13.31, 95% CI:5.13, 34.53).

**Conclusions:**

The common under-five childhood illnesses were acute respiratory tract infection and diarrhea. The study also revealed that there was a delay in seeking healthcare and this was significantly associated with age of the child; mothers/caregivers level of education; family size; perception of illness severity; previous experience of similar illnesses and under-five child death.

**Electronic supplementary material:**

The online version of this article (10.1186/s12887-019-1588-2) contains supplementary material, which is available to authorized users.

## Background

Children are the most vulnerable age group in any community; hence, the under-five mortality rate (U5MR) is a widely used demographic measure and an important indicator of the level of welfare in countries [1]. In this regard, considerable achievement has been made towards decreasing U5MR, where it has declined nearly by 50% globally between 1990 and 2015 [2] and by 60% in Ethiopia between 2000 and 2016 [3]. In the face of these gains, however, half a million children are dying each year from easily preventable diseases [4].

Globally, more than half of early childhood complications and deaths are due to ill health that can be prevented or treated with simple and affordable interventions [5, 6]. In sub-Saharan Africa, 1 in 12 child dies before celebrating the fifth birthday [2, 7] and 1 in 11 Ethiopian child dies before the fifth birthday [8]. Infectious diseases turn out to be the most common causes of child morbidity and mortality in most developing countries; Ethiopia being the forefront [5, 7]. The top five leading cause of morbidity in Ethiopia for children under-five years are infectious diseases; diarrhea (20%), pneumonia (19%), acute respiratory infections (ARI) (15%) and acute febrile illnesses (AFI) (7%) [3]. Furthermore, they are major causes of under-five mortality as well as inpatient admissions [9].

Studies suggest that common causes of under-five morbidity and mortality in developing countries could substantially be reduced with timely healthcare seeking behavior (HCSB) of their families [10]. On the contrary, studies substantiate that a large number of sick children do not visit health facilities [11]. This means that most children die without ever reaching a health facility and due to delays in seeking healthcare [5].

Healthcare seeking behavior is not only because of availability of health facilities and other sources of healthcare but also motivation and ability of individuals to seek medical care. Seeking healthcare in children is unique as parents decide upon the type and frequency of healthcare service accessed [12]. Accordingly, a number of factors can influence mother’s HCSB for their sick children. Poor socio-economic status, attitude to modern treatment, low literacy level of the parents, large family size, and number of symptoms, previous experience of child illness and death, and perceived severity of illness were the most commonly mentioned factors affecting HCSB [5]. First and foremost, mothers in developing countries usually do not have sufficient knowledge to recognize danger signs or what appropriate treatment should be to their child health [10]. Secondly, millions of mothers and their children live in remote areas, where the social environment is against seeking healthcare [6]. In such social context, peoples choose home/self-treatment, use of traditional treatment above modern healthcare services [5, 13]. Thirdly, parental belief and anticipation about the outcome of therapy was identified as a barrier or early termination in seeking treatment for their children [5].

Improving families HCSBs for their children can contribute to significantly reduce morbidity and mortality of under-five children. According to World Health Organization (WHO) estimate, seeking timely and appropriate healthcare could reduce child deaths due to ARIs by 20%. Similarly, in 2016 Ethiopian Demographic and Health Survey (EDHS), prompt medical attention was crucial in reducing child deaths [3]. The risk of death from common childhood illnesses when complicated is greatest and thus it has been established that early diagnosis and prompt treatment should occur within 24 h of the onset of these illnesses [10]. In addition, effective management of childhood illness necessitates a partnership between families and health workers. Maternal practices regarding child healthcare have been recognized as the main factor behind preventing morbidities and mortality among children [14]. This suggests that a proper understanding of mothers/caregivers HCSB may reduce delay in diagnosis, improve treatment compliance and improve health promotion strategies in a variety of contexts [15]. Therefore, this study aimed to assess HCSB of mothers/caregivers towards childhood illnesses in selected health centers of Addis Ababa, Ethiopia.

## Methods

### Study design and setting

A facility-based cross-sectional study was conducted in Addis Ababa, Ethiopia between April 18 to May 11, 2016. Administratively Addis Ababa is divided into 10 sub-cities and it has a total of 56 hospitals (14 of which are public) and 96 health centers [16]. The city administration has a total population of 3,352,000; where 7.16% of them are children under-five years old [9]. All mothers/caregivers who reside in Addis Ababa and have at least one child who is under-five years of age with childhood illnesses formed the study populations. Those mothers/caregivers with a child who is under-five years of age and visited the selected facilities to seek healthcare for childhood illnesses were included in the study. However, mothers/caregivers were excluded, if their purpose of the visit was for follow-up of their child, a child presented with signs and symptoms necessitating urgent referral. The dependent variable is HCSB of mothers/caregivers and the independent variable comprises factors: age of mother/caregiver, age of child and gender, family size, level of education of mother/caregiver, occupation of mother/caregiver, marital status, birth order, average monthly family income, perception of severity of child illness, number of symptoms,; service utilized by the mother and the child, previous experiences of child illness, previous experience of under-five death, and duration of illness.

### Sample size and sampling techniques

The sample size was determined by using single proportion formula [17]. The prevalence of healthcare seeking behavior of mothers/caregivers for childhood illnesses in Addis Ababa was unknown and for this study, 50% prevalence was used with the intention of getting the maximum sample size. Thus, considering 5% margin of error, 95% confidence level and 10% contingency, the total sample size to be included in the study was 422. A total of five sub-cities and ten health centers; two health centers from each sub-city (Kotebe, Yeka, Shiromeda, Hidasse, Lomimeda, Keranio, Arada, Churchill, Woreda amest and Woreda hulet health centers) were selected using simple random sampling method by employing lottery method. Then, proportionate number of mothers/caregivers were recruited from the selected health centers. Finally, the number of mothers/caregivers to be recruited from each health center was decided based on the proportion to the size of clients attended in under-five outpatient department from the previous one year data. Whenever there were more than one under-five year’s old children brought by the mother/caregiver, one of them was selected using lottery method.

### Data collection procedures

Data were collected using a pre-tested, semi-structured questionnaire consisting of 33 questions (Additional file 1). The questionnaire was designed in a way to capture socio-demographic or economic characteristics and questions related to mothers/caregivers HCSB and its associated factors. It was developed by reviewing previous works of literature [1, 12, 18] and modified to the study context. It was initially prepared in English and then translated into local language, Amharic and then re-translated into English to check its consistency.

Two health officers and five clinical nurses were recruited as supervisors and data collectors, respectively. To avoid potential bias, it was ensured that data collectors were not working in the under-five outpatient department as well as in the sampled health centers. They attended a one-day training focused on the aim of the study and detailed review of the tools. Besides the practical training, adequate supervision and follow up were done by the supervisors to maximize the quality of the data collected.

### Data processing and analysis

Data were checked for completeness, coded and entered into EPI-info version 3.5.4 and exported into SPSS version 20.0 for cleaning and statistical analysis. Descriptive statistics like graphs, tables, frequency, percentage and standard deviation were computed to describe the events. The presence and strength of association of outcome variable were assessed using multivariable logistic regression. After performing the bivariate analysis, all variables with *p*-value < 0.25 were considered for the multivariable analysis. Significant associations between variables were determined at *p*-value < 0.05.

For this study, healthcare seeking behavior is defined as mothers/caregivers reaching to the health service within 24 h of the illness of the child.

Ethical clearance was obtained from Addis Ababa Public Health Research and Emergency Control Office (Approval Number: A/A/H/7072/227) and as per the permission of the ethics committee, informed verbal consent to participate was obtained from all mothers/caregivers. Prior to data collection, the data collectors explained the purpose of the study in Amharic (local language) and read to them an informed consent script which was approved by the ethics committee. Participants were also assured of the confidentiality and anonymity of the information obtained. Permission to conduct the study was also sought from the selected health facilities. Collected questionnaires were coded and locked in a lockable cabinet in order to maintain confidentiality of the information obtained.

## Results

### Socio-demographic characteristics of respondents

A total of 422 mothers/caregivers with a child under-five years old were interviewed, with a response rate of 100%. Of these, 361(85.5%) were mothers as primary caregiver. Mean age of mothers/caregivers was 28.8 ± 5.31 years, ranged from 18 to 58 years. Three hundred sixty three (86.0%) of mothers/caregivers were married; more than half of them (57.2%) had a secondary educational level and 183(43.4%) had no occupation (Table 1).

**Table 1:** Socio-demographic characteristics of child and mothers/caregivers in Addis Ababa, Ethiopia

Variables	Frequency (n)	Percentage (%)
Primary caregiver		
Mother	361	85.5
Father	49	11.6
Others (Grandmother, Aunt)	12	2.9
Age of primary caregiver		
≤ 28 years	223	52.8
> 28 years	199	47.2
Marital Status of primary caregiver		
Married	363	86.0
Not married (Single, Divorced, Widowed)	59	14.0
Educational status of primary caregiver		
No school	50	11.8
Primary school (Grade 1–8)	33	7.8
Secondary school (Grade 9–12)	241	57.2
Tertiary (Diploma and above)	98	23.2
Occupation of primary caregiver		
No occupation	183	43.4
Government employee	91	21.6
Private employee	55	13.0
Self-employee	93	22.0
Average monthly family income (ETB)		
< 1300	99	23.5
1301–2800	113	26.8
2801–4700	112	26.5
4701–7000	73	17.3
> 7000	25	5.9
Total number of family size		
≤ 5	372	88.2
> 5	50	11.8
Gender of the child		
Boy	205	48.6
Girl	217	51.4
Age of the child in months		
≤ 12 months	138	32.7
13–60 months	284	67.3
Birth order of the child		
First	216	51.2
Second	139	32.9
Third	53	12.6
Fourth and Above	14	3.3
Number of children		
One child	328	77.7
Two or more children	94	22.3

Three hundred seventy two (88.2%) of the mothers/caregivers were from households having ≤5 family size. The mean number of family size was 3.88 ± 1.21, ranged from 1 to 11. More than half (51.2%) of the mothers reported that it was their first child. The mean age of the children was 25 ± 16.5 months, ranged from 1 to 59 months (Table 1).

### Magnitude, previous experience and severity perception of childhood illness

**Fig. 1:**
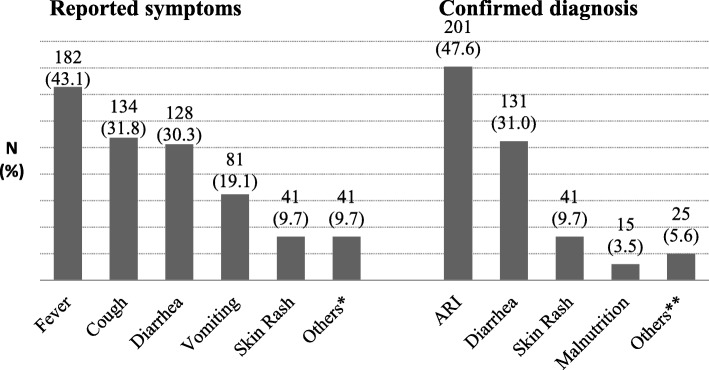
^a^Symptoms observed by mothers/caregivers and confirmed diagnosis in Addis Ababa, Ethiopia. ^a^Numbers do not add up to 422 because, because a child has more than one symptom as well as diagnose. *Eye diseases, Burning sensation upon urination, Ear pain/discharge, Tooth ache, Discharge from the umbilicus. **Conjunctivitis, Urinary Tract Infection, Otitis media, Dental Caries, omphalitis, Measles, Asthma

The diseases specific magnitude of most common symptoms reported by the mothers/caregivers were fever, cough, diarrhea and vomiting among 182(43.1%), 134(31.8%), 128(30.3%), and 81(19.2%) of the children, respectively. The average symptom days reported was 3 ± 4.0 days, ranging from 1 to 36 days. From the result, the commonest childhood diseases that were confirmed by health professionals for under-five children were ARI and diarrhea, which accounts for 201(47.6%) and 131(31.0%), respectively (Fig. 1). With regard to mothers/caregivers perception of illness severity, 128(30.3%) of them perceived it as severe, 163(38.6%) as moderate and 131(31.1%) as mild.

Thirty eight (9.0%) mothers/caregivers reported that the index child had an experience of similar type of illness previously. Of the 94 mothers/caregivers, who had two or more children, 39(9.2%) of them reported a previous history of under-five child death in the family, of which 43.6% of them died after completing treatment, 48.7% of deaths occurred while the child was on treatment and the rest (7.7%) did not seek any treatment prior to the event (Table 2).

**Table 2:** Mothers/caregivers previous experiences and actions taken for the sick child, Addis Ababa, Ethiopia

Variables	Frequency (n)	Percentage (%)
Previous experience of similar illnesses		
Yes	38	9.0
No	384	91.0
Previous experience of under-five child death		
Yes	39	9.2
No	55	90. 8
If yes, what was the possible cause of death		
Due to illness and after treatment	17	43.6
Due to illness and on treatment	19	48.7
Due to illness and without no any treatment	3	7.7
Possible reasons not to take the child to health facility within 24 h*		
Mild illnesses	150	35.5
Self-Limiting	149	35.3
Lack of time	25	5.9
Shortage of money	24	5.7
Others^a^	26	6.16
Action taken within 24 h		
I treat the child at home with safe homemade remedy	138	32.7
I treat the child with available modern medicine at home	80	19.0
I purchase modern medicine from community pharmacy	76	18.0
Others ^b^	16	3.8

Note: Others ^a:^ - Mothers/caregivers perceived illnesses is due to teething so, waited till the illness gets improved; took to community pharmacy; thought the illnesses is not curable

Others ^b:^ - Took to religious place; traditional healers

*Numbers do not sum up to 310 because answers were with multiple responses

### Healthcare seeking behavior of mothers/caregivers for their sick child

**Fig. 2:**
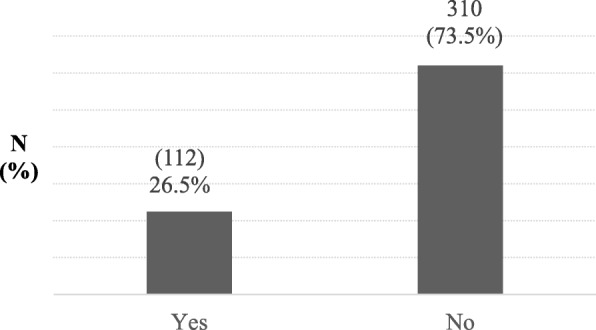
Healthcare sought within 24 h of recognition of illness in Addis Ababa, Ethiopia

One hundred twelve (26.5%) of the mothers/caregivers sought healthcare for their sick child on the first day of recognition of illness. On the other hand, 310(73.5%) sought health care one or two days after the recognition of illnesses (Fig. 2). The main reasons for those who came to health facility later than 24 h of recognition of illnesses were, illness was considered as mild for 150(35.5%) mothers/caregivers, self-limiting 149(35.3%), and the rest were due to shortage of money and lack of time. Furthermore, from the 310 respondents who didn’t take their child to the health facility within 24 h, 138(44.5%) of them had attempted to treat their child at home by giving only safe homemade remedies and 80(25.8%) treated with hoarded modern medicines. A quarter (24.5%) of treated their child by purchasing modern medicine from community pharmacies (Table 2).

### Mothers/caregivers healthcare seeking behavior towards childhood illness

Child gender, duration of illness and service utilized by the mother and child were excluded from the multivariable analysis since they had had *p*-value > 0.25. Multivariable logistic regression analysis showed that age of the child, education of mothers/caregivers, number of family member, perception of severity of the illnesses, previous experience of similar illnesses, and previous history of under-five child death in the family were significantly related with HCSB of mothers/caregivers for childhood illnesses. However, the age of mothers/caregivers, marital status of mothers/caregivers, occupational status of mothers/caregivers, number of symptoms were not significantly associated with mothers/caregivers HCSB (Table 3).

**Table 3:** Factors associated with mothers/caregivers healthcare seeking behavior towards childhood illnesses, Addis Ababa, Ethiopia

Variables	Reach to health facility within 24 h		Odds Ratio (95% CI)	
	Yes; n (%)	No; n (%)	Crude	Adjusted
Child Age (in months)				
1–12	55 (13.0)	83 (19.7)	2.63 (1.68, 4.13)*	1.78 (1.02, 3.13)*
13–60	57 (13.5)	227 (53.8)	1.00	1.00
Birth Order				
First	64 (15.2)	152 (36.0)	1.00	1.00
Second	31 (7.3)	108 (25.6)	0.68 (0.41, 1.11)	0.51 (0.26, 1.00)
Third	13 (3.1)	40 (9.5)	0.77 (0.38, 1.54)	0.76 (0.29, 1.96)
Fourth and above	4 (0.9)	10 (2.4)	0.95 (0.28, 3.14)	3.16 (0.52, 19.06)
Age of primary care giver				
≤ 28	72 (17.0)	151 (35.8)	1.89 (1.21, 2.96)*	1.81 (0.97, 3.37)
> 28	40 (9.5)	159 (37.7)	1.00	1.00
Marital Status				
Married	100 (23.7)	263 (62.3)	1.48 (0.75, 2.92)	1.43(0.59, 3.48)
Currently not married	12 (2.8)	47 (11.2)	1.00	1.00
Education				
No education	4 (1.0)	46 (10.9)	1.00	1.00
Primary school	6 (1.4)	27 (6.4)	2.55 (0.66, 9.87)	1.77(0.39, 8.01)
Secondary school	70 (16.6)	171(40.5)	4.70 (1.63,13.57)*	4.24 (1.32, 13.63)*
Tertiary school	32 (7.6)	66 (15.6)	5.57 (1.84,16.84)*	3.48 (0.88, 13.63)
Occupation				
No occupation	43 (10.2)	140 (33.2)	1.00	1.00
Government employee	33 (7.8)	58 (13.8)	1.85 (1.07, 3.20)*	2.04 (0.90, 4.58)
Private employee	16 (3.8)	39 (9.2)	1.33 (0.68, 2.62)	1.35 (0.56, 3.25)
Self-employee	20 (4.7)	73 (17.3)	0.89 (0.48, 1.62)	1.30 (0.62, 2.72)
Family size				
≤ 5 members	106 (25.1)	266 (63.0)	2.92 (1.20,7.06)*	3.83 (1.06, 13.78)*
> 5 members	6 (1.4)	44 (10.5)	1.00	1.00
Number of symptoms				
One symptom	62 (14.7)	205 (48.6)	1.00	1.00
Two or more symptoms	50 (11.8)	105 (24.9)	1.57 (1.01, 2.44)*	1.19 (0.66, 2.14)
Perception of severity of illness				
Sever	60 (14.2)	68 (16.1)	4.10 (2.59, 6.49)*	2.00 (1.05, 3.84)*
Not sever	52 (12.3)	242 (57.3)	1.00	1.00
Previous experience of child illnesses				
Yes	20 (4.7)	18 (4.3)	3.52 (1.78, 6.95)*	3.67 (1.36, 9.86)*
No	90 (21.8)	292 (69.2)	1.00	1.00
Previous history of Under-five Death				
Yes	28 (29.8)	11 (11.7)	9.06 (4.33,18.95)*	13.31 (5.13,34.53)*
No	12 (12.8)	43 (45.7)	1.00	1.00

*significant at *p*-value < 0.05

The study revealed that mothers/caregivers who had a child less than or equal 12 months were two times more likely to seek healthcare than those having greater than 12–60 months (AOR = 1.78, 95% CI:1.02, 3.13). The study also showed that there is a statistically significant association of educational status of mothers/caregivers and HCSB, where mothers/caregivers with secondary level of school had nearly four times more likely to seek healthcare as compared with those who had no education (AOR = 4.24, 95% CI:1.32, 13.63) (Table 3).

Regarding with the number of family size, a family member that has less than or equal to five members were four times more likely to seek healthcare when compared to those having greater than five members (AOR = 3.83, 95% CI:1.06, 13.78). Moreover, mothers/caregivers who perceived their child’s illness as sever were two times more likely to seek healthcare compared to those who perceive the illness as not sever (moderate or mild) (AOR = 2.00, 95% CI:1.05, 3.84). The likelihood of seeking healthcare among children was 3.6 times higher for those mothers/caregivers who had an experience of previous illnesses (AOR = 3.67, 95% CI:1.36, 9.86). Mothers/caregivers with a previous experience of under-five death were 13 times more likely to seek healthcare compared to those who had no history of child death (AOR = 13.31, 95% CI:5.13, 34.53) (Table 3).

## Discussion

This study investigated the magnitude and determinants of mothers/caregivers HCSB towards childhood illnesses in the sampled health centers of Addis Ababa, Ethiopia. Almost half of the under-five children in this study had ARI and 31.0% of the children had diarrhea which is comparable to finding in Debrebirehan referral hospital (31.7%) [19]. The high prevalence of diarrhea could be attributed to the short term rainy season during the data collection period which might have degraded surface and ground water microbial quality. As compared to 2011 EDHS findings, which has reported 70% ARI and 13% Diarrhea prevalence, our study highlights decreasing ARI occurrence and increasing diarrhea [8]. The possible discrepancy in the prevalence could be because EDHS’s study was based on national samples including the rural settings.

The findings showed that mothers/caregivers who had a child younger than 12 months were more likely to seek healthcare for their child on the first day of onset of illness as compared to a child above 12 months. This might be because mothers/caregivers understand that younger children have lower immunity than their elder siblings [20].

In this result, families that have less than or equal to five members were four times more likely to seek healthcare for the sick child when compared to those having greater than five members. This shows that children born from larger family size are also less likely to get immediate care from health facilities. Likewise, results in Tanzania showed that children from households having two to three under-five children were more likely to receive medical care late than those from households which had only one under-five child [21]. Studies showed that mothers/caregivers high workload due to large family size bring about giving lesser attention to the sick child. Furthermore, financial restraints of large family members to visiting health facilities was related to family size [6, 22].

The study revealed that mothers/caregivers with secondary school education were four times more likely to seek healthcare than non-educated ones. This implies that the higher the level of education, the better the HCSB. This might be due to the fact that educated mothers are more likely to be able to read thereby, understand better and adopt practices for a preventive and curative child as promoted through health education in outreach programs or by healthcare providers. Mother’s education can also be associated with hygiene and HCSBs relevant to decrease childhood illness [23, 24].

Similarly, according to findings of other studies, educated mothers/caregivers are associated with higher odds of seeking treatment [1, 5, 25]. Hence, it is crucial to improve awareness of mothers/caregivers about common childhood illnesses through enhancing mothers/caregivers level of education.

This result showed that 26.5% of mothers/caregivers sought healthcare for their sick child within 24 h of recognition of illnesses. This means that healthcare seeking in a health facility is delayed. Comparably, result in North Shoa, Ethiopia, showed experience of seeking healthcare by mothers/caregivers were only 13.5% on the first day of onset of illness of their sick child [6]. Their tendency to try home remedies; lack of money; and their thought that the illness was mild or will resolve by its self were mentioned as some of the reasons for a delay in seeking healthcare [1, 25].

The most frequent first action taken by mothers/caregivers who did take their child later than 24 h of recognition of illness was to treat the child at home. Such mothers/caregivers seem to prefer household treatments rather than making use of professional medical services as a trusted source of care, instead of taking it as the last solution after trying other trusted methods, which is a perilous situation [1]. Alike this, result in South Africa found that, treatment by home remedies was the most common first action by caretakers for children [26]. Similarly, finding in Yemen as well as Ethiopia revealed that households’ treatments were the main source of treatment [1, 27].

In this study, it was fascinating to note that nearly 40% of mothers/caregivers ‘treated their child with leftover medicines or un-prescribed modern medicine that was bought from a community pharmacy for their ill child. In many areas, modern pharmaceutical agents are a commonly misused as a first-line therapy for the home treatment of childhood illnesses or they serve as an alternative when traditional remedies fail [28]. Comparably in Nepal, 61.3% of episodes of childhood illnesses were treated with medicines that were purchased from the pharmacies without consultation [29]. Similarly, a study in Yemen reported that most frequent first response was purchasing over-the-counter medicines [1]. This fact that many families cannot afford to perform the tests doctors recommend and/or busy parents prefer to purchase medicine from the nearest pharmacy are contributing to the event [29]. Unless regulated, this irrational use of medicines will contribute to increasing undesirable consequences of medicines like the development of antibiotic resistance [30]. Hence, strong regulatory enforcement should be in place to prohibit over-the-counter sales of prescription-only-medicines from the community pharmacies and efforts should be made to encourage families to promptly visit health facilities.

This study revealed that mothers/caregivers decision to manage child illness was influenced by their perception of its severity which is consistent with the study conducted in Derra District, Ethiopia [6]. It is also true that people use first what is most easily available to them, but when the illnesses is serious, they start to make a greater effort in order to try something better [1]. The present study also showed that mothers/caregivers who had a history of under-five deaths were more likely tend to seek treatment earlier study conducted by Getahun et al (2010) in south-west Ethiopia [31]. This implies that perceived risk and severity of the illness as well as, prior bad experience can influence the initiation of HCSB. One possible reason is that mothers who have experienced a child death in the past might be more sensitive to trying to save another child’s life [32].

## Conclusions

Acute Respiratory Infection and diarrhea were the most common childhood illnesses in under-five children. The study also showed that there was a delay in seeking healthcare to the sick children. Age of the child, mothers/caregivers level of education, number of family size, perception of the severity of illness, previous experience of similar illnesses and under-five child death were associated to mothers/caregivers decision to seek health care for their sick child.

## Additional file

Additional file 1: Quantitative survey questionnaire. (DOCX 34 kb)

## Data Availability

The datasets used and/or analyzed during the current study are available from the corresponding author on reasonable request.

## Abbreviations

AFI: Acute Febrile Illnesses
ARI: Acute Respiratory Infection
CSA: Central Statistical Agency
EDHS: Ethiopian Demographic and Health Survey
FMOH: Federal Minister of Health
HCSB: Healthcare seeking behavior
