# Supplementary material for: Mothers/caregivers healthcare seeking behavior towards childhood illness in selected health centers in Addis Ababa, Ethiopia: a facility-based cross-sectional study
Source: BMC Pediatr. 2019 Jul 3;19:220. doi: 10.1186/s12887-019-1588-2 (PMC6607537; doi:10.1186/s12887-019-1588-2)
Supplement: Supplementary file 1 — Quantitative survey questionnaire. (DOCX 34 kb) [file 12887_2019_1588_MOESM1_ESM.docx]

English versions of information sheet, consent form and questioner designed to assess factors determining primary caregivers' healthcare-seeking behavior towards common childhood illnesses.

**Information given to study participant before their participation in the study**

My name is_______________. I am here on the behalf of Nebiat Teshager, a post graduate student from Africa Medical College, currently carrying out a research on factors determining mothers/caregivers healthcare-seeking behavior towards common childhood illnesses in the sampled health centers, in Addis Ababa, Ethiopia. The study will provide information that might enable health personnel, government and community to improve healthcare-seeking behavior for childhood illness. The appropriate authorities have approved the study. I would like to take your time to respond to this interview questions and it will take approximately 30 – 40 minutes. I kindly request you to answer as truthfully as possible. Your answer will not be revealed to any other people, and the information you give will be treated anonymously and confidential. This research imposes no risk and therefore no compensation will be provided for your participation in this study. Your participation is voluntary, you can withdraw anytime or refuse to continue, and this will not influence the way you will be treated in the health institution or in the community.

**Informed consent**

I have been explained all information and procedures that are part of this research study and I have understood. I understand that the research imposes no risk on my life and therefore no compensation would be provided. I hereby agree to participate in this research study and give my voluntary consent. I hereby also give rights to the researcher for collecting the data that are required for the study.

Agree Disagree

Name of the Interviewer____________________ Signature__________ Date ___________

Name of the Supervisor_____________________ Signature__________ Date ___________

Researcher: Nebiat Teshager

Phone No: +251 913 28 0529

Email: [nteshager2@gmail.com](mailto:nteshager2@gmail.com)

Code No._____________ Sub city ____________Kebele______________

***Questioners in English version on factors determining mothers/caregivers healthcare-seeking behavior for common childhood illnesses in Addis Ababa, Ethiopia***

| Section 1: General information on socio-demographic and socio-economic characteristic of mothers/caregivers and the child | | | | |
| --- | --- | --- | --- | --- |
| Encircle the answer of your choice on the numbers before the answers | | | | |
| S. N^o^ | Questioner | Answer | | Skip |
| 101 | Age of the child | ____________________ Month | |  |
| 102 | Sex of the child | 1. Boy 2. Girl | |  |
| 103 | Birth order of the child | 1. First child 2. Second child 3. Third child 4. Other(Please specify_______________ ) | |  |
| 104 | Relationship to the child | 1. Mother 2. Father 3. Care giver 4. Other (Please specify_______________ ) | |  |
| 105 | Age of mother/caregiver | ________________________ years | |  |
| 106 | Residence of mother / caregiver | 1. Addis Ababa  2. Outside Addis Ababa, (Please mention___) | |  |
| 107 | The highest educational level of mother / caregiver | 1. No school 2. No formal education (Read & write only) 3. Grade 1-4 4. Grade 5-8 5. Grade 9-10 6. Grade11-12 7. Technique and vocational / Diploma 8. Degree and above | |  |
| 108 | Occupation of mother | 1. House wife 2. Government employee 3. Private employee 4. Merchant 5. Self-employee 6. Other(Please specify _______________ ) | |  |
| 109 | Average monthly income of mother / caregiver | _______________________ ETB | If no income please say 'NA' | |
| 110 | Average monthly income of the family | ______________________ ETB | If no income please say 'NA' | |
| 111 | Marital status of the mother | 1. Single 2. Married 3. Divorced 4. Widowed | If answer is other than married skip to Q# 114 | |
| 112 | The highest educational level of the father? | 1. Illiterate. 2. Read and write 3. Grade 1-4 4. Grade 5-8 5. Grade 9-10 6. Grade 11-12 7. Technique and Vocational ET/ Diploma 8. Degree and above | |  |
| 113 | Fathers' occupation | - - 1. No occupation     2. Government employee     3. Private employee     4. Merchant     5. Self-employee     6. Other(Please specify _______________ ) | |  |
| 114 | Number of family members living in the house. | 1. Children under five years _____ 2. Total number of children _____ 3. Total number of family _______ | If total n^o^ of children is equal to one skip to Q # 116 | |
| 115 | If mother has more than one child, what is the average birth interval between the siblings? | 1. One year 2. Two years 3. Three years 4. Other(Please specify ______________ ) | |  |
| 116 | Is there any under-five death in the family | 1. Yes 2. No | If No, skip Q # 119 | |
| 117 | If yes, what was the possible cause of death | 1. Due to illness and after treatment 2. Due to illness and on treatment 3. Due to illness without any treatment 4. Other (Please specify _____________ ) | |  |

| Section 2: HCSBs of mothers/caregivers for common childhood illness | | | |
| --- | --- | --- | --- |
| Encircle the answer of your choice on the numbers before the answers | | | |
| S. N^o^ | Question | Answer | Remark |
| 201 | What kind of symptom(s) have you observed on your child? | 1. Cough 2. Difficulty of breathing 3. Fever 4. Diarrhea 5. Vomiting 6. Skin rash 7. Discharge from the eye 8. Other (Please specify _______ ) | More than one answer is possible |
| 202 | If the child has fever, what is the temperature level (measured by axillary) | ___________________^o^ C |  |
| 203 | Duration of illnesses before treatment was sought | ____________________ Days |  |
| 204 | Did you reach to the health facility within 24 hours from the recognition of illness | 1. Yes 2. No | If yes, skip Q# 204 |
| 205 | If mother/caregiver did not reach to the health facility /sought medical care within 24 hours, what could be the possible reason to delay in seeking medical care? | 1. I thought the illness was self-limiting / waiting for illness to subside/no action taken 2. I thought the illness was mild. 3. I have shortage of money. 4. I do not have enough time. 5. I thought illness was incurable. 6. I do not know where it can be treated 7. Transportation difficulty 8. Other (Please specify _____________ ) |  |
| 206 | What is the confirmed diagnosis of your child by health professional? | ___________________ | (Please fill this from patient card) |
| 207 | What action did you take first when you observe this symptom(s), before you bring the child to this health center? | 1. First, I brought the child here, without any previous intervention (Do nothing). 2. First, I treat the child at home with safe homemade remedy(like honey, milk …) 3. First, I treat the child with avaliable modern medicine at home. 4. First, I purchased modern medicine from a community pharmacy 5. First, I took the child to a private health facility. 6. First, I took the child to a religious place. 7. First, I took the child to traditional healers. 8. Others (Please specify ______________ ) |  |
| 208 | If your answer to question number 206 is "purchased modern medicine from a community pharmacy" what was the reason to purchase modern medicine from pharmacy? | 1. I thought the illness was not severe. 2. The pharmacy was near. 3. I thought there is high cost for medical visit/services. 4. I thought they order unnecessary laboratory investigation. 5. Other (Please specify ________________ ) |  |
| 209 | If your answer to question number 206 is traditional healer, what was the reason to go to traditional healers? | 1. I thought there is no cure for this disease. 2. I thought they do not charge too much / lack of money. 3. I had a previous good experience in traditional healers. 4. I had a previous bad experience in modern medical care. 5. I do not have trust (poor attitude) in health care providers. 6. Others (Please specify _______________ ) |  |
| 210 | According to your perception, how sever was the illness of the child? | 1. Severe 2. Moderate 3. Mild | If illness is not sever skip Q# 211 |
| 211 | If you think the child is severely ill, how do you identify the severity of the illness? | 1. The child has one or more of the symptoms (convulsion, loss of consciousness, unable to breast feed, vomits everything). 2. The child has cough 3. The child has difficulty of breathing 4. The child has fever 5. The child has diarrhea 6. The child refuses to eat/ loss of appetite. 7. The child stops playing. 8. Symptom(s)did not get relief, it gets worse 9. From previous of similar illnesses 10. Other people told me so. 11. Other (Please specify ______________ ) |  |
| 212 | Who is the decision maker to take the child for medical treatment when the child is ill? | 1. Mother 2. Father 3. Both (Mother and Father) 4. Caregiver. 5. Other (Please specify _______________ ) |  |
| 213 | Who brings the child to the health facility today? | 1. Mother 2. Father 3. Caregiver 4. Other (Please specify _______________ ) |  |
| 214 | Did the mother have at least one ANC or PNC follow-up? | 1. Yes. 2. No. |  |
| 215 | Place of birth of the child? | 1. Government health facility 2. Private health facility 3. Home delivery |  |
| 216 | Immunization status of the index child | 1. Fully immunized 2. Immunization updated. 3. Discontinued Immunization 4. Not immunized at all |  |

End of the interview, Thank you
